# Supplementary material for: Pyruvate Dehydrogenase Kinase 1 inhibition mediated oxidative phosphorylation enhancement in cartilage promotes osteoarthritis progression
Source: BMC Musculoskelet Disord. 2023 Jul 20;24:597. doi: 10.1186/s12891-023-06585-6 (PMC10357736; doi:10.1186/s12891-023-06585-6)
Supplement: Supplementary file 1 — Additional file 1. [file 12891_2023_6585_MOESM1_ESM.zip › MF.Gsea.1653627316242/GOMF_FRIZZLED_BINDING.html]

Details for gene set GOMF\_FRIZZLED\_BINDING[GSEA]

|  || Dataset | 55457\_collapsed\_to\_symbols.cls.cls #OA\_versus\_normal.cls.cls #OA\_versus\_normal\_repos |
| Phenotype | cls.cls#OA\_versus\_normal\_repos |
| Upregulated in class | OA |
| GeneSet | GOMF\_FRIZZLED\_BINDING |
| Enrichment Score (ES) | 0.31838375 |
| Normalized Enrichment Score (NES) | 1.1440126 |
| Nominal p-value | 0.2525 |
| FDR q-value | 0.61624396 |
| FWER p-Value | 1.0 |
Table: GSEA Results Summary

  

Fig 1: Enrichment plot: GOMF\_FRIZZLED\_BINDING      
 Profile of the Running ES Score & Positions of GeneSet Members on the Rank Ordered List

  

| SYMBOL | TITLE | RANK IN GENE LIST | RANK METRIC SCORE | RUNNING ES | CORE ENRICHMENT || 1 | FZD1 | frizzled class receptor 1 [Source:HGNC Symbol;Acc:HGNC:4038] | 111 | 0.898 | 0.0840 | Yes |
| 2 | FZD7 | frizzled class receptor 7 [Source:HGNC Symbol;Acc:HGNC:4045] | 274 | 0.768 | 0.1506 | Yes |
| 3 | WNT5B | Wnt family member 5B [Source:HGNC Symbol;Acc:HGNC:16265] | 327 | 0.734 | 0.2222 | Yes |
| 4 | CCDC88C | coiled-coil domain containing 88C [Source:HGNC Symbol;Acc:HGNC:19967] | 1455 | 0.435 | 0.1795 | Yes |
| 5 | MYOC | myocilin [Source:HGNC Symbol;Acc:HGNC:7610] | 1765 | 0.389 | 0.1955 | Yes |
| 6 | WNT5A | Wnt family member 5A [Source:HGNC Symbol;Acc:HGNC:12784] | 1995 | 0.361 | 0.2150 | Yes |
| 7 | WNT3 | Wnt family member 3 [Source:HGNC Symbol;Acc:HGNC:12782] | 2003 | 0.360 | 0.2516 | Yes |
| 8 | ROR2 | receptor tyrosine kinase like orphan receptor 2 [Source:HGNC Symbol;Acc:HGNC:10257] | 2448 | 0.308 | 0.2489 | Yes |
| 9 | SFRP1 | secreted frizzled related protein 1 [Source:HGNC Symbol;Acc:HGNC:10776] | 2503 | 0.303 | 0.2759 | Yes |
| 10 | DVL2 | dishevelled segment polarity protein 2 [Source:HGNC Symbol;Acc:HGNC:3086] | 2693 | 0.280 | 0.2901 | Yes |
| 11 | WNT2 | Wnt family member 2 [Source:HGNC Symbol;Acc:HGNC:12780] | 2700 | 0.279 | 0.3184 | Yes |
| 12 | WNT2B | Wnt family member 2B [Source:HGNC Symbol;Acc:HGNC:12781] | 4194 | 0.138 | 0.2167 | No |
| 13 | DVL1 | dishevelled segment polarity protein 1 [Source:HGNC Symbol;Acc:HGNC:3084] | 4612 | 0.104 | 0.1950 | No |
| 14 | WNT11 | Wnt family member 11 [Source:HGNC Symbol;Acc:HGNC:12776] | 4688 | 0.097 | 0.1991 | No |
| 15 | WNT6 | Wnt family member 6 [Source:HGNC Symbol;Acc:HGNC:12785] | 5781 | 0.010 | 0.1153 | No |
| 16 | RYK | receptor like tyrosine kinase [Source:HGNC Symbol;Acc:HGNC:10481] | 5896 | 0.002 | 0.1067 | No |
| 17 | WNT7B | Wnt family member 7B [Source:HGNC Symbol;Acc:HGNC:12787] | 6230 | -0.023 | 0.0831 | No |
| 18 | RNF43 | ring finger protein 43 [Source:HGNC Symbol;Acc:HGNC:18505] | 6609 | -0.050 | 0.0589 | No |
| 19 | WNT7A | Wnt family member 7A [Source:HGNC Symbol;Acc:HGNC:12786] | 7166 | -0.093 | 0.0253 | No |
| 20 | NDP | norrin cystine knot growth factor NDP [Source:HGNC Symbol;Acc:HGNC:7678] | 7631 | -0.129 | 0.0026 | No |
| 21 | WNT10B | Wnt family member 10B [Source:HGNC Symbol;Acc:HGNC:12775] | 7700 | -0.134 | 0.0111 | No |
| 22 | BAMBI | BMP and activin membrane bound inhibitor [Source:HGNC Symbol;Acc:HGNC:30251] | 7903 | -0.148 | 0.0106 | No |
| 23 | DVL3 | dishevelled segment polarity protein 3 [Source:HGNC Symbol;Acc:HGNC:3087] | 8756 | -0.213 | -0.0336 | No |
| 24 | WNT16 | Wnt family member 16 [Source:HGNC Symbol;Acc:HGNC:16267] | 8808 | -0.218 | -0.0152 | No |
| 25 | WNT1 | Wnt family member 1 [Source:HGNC Symbol;Acc:HGNC:12774] | 10706 | -0.394 | -0.1220 | No |
| 26 | DLG4 | discs large MAGUK scaffold protein 4 [Source:HGNC Symbol;Acc:HGNC:2903] | 11077 | -0.441 | -0.1052 | No |
| 27 | WNT8B | Wnt family member 8B [Source:HGNC Symbol;Acc:HGNC:12789] | 11532 | -0.505 | -0.0884 | No |
| 28 | SDCBP | syndecan binding protein [Source:HGNC Symbol;Acc:HGNC:10662] | 12008 | -0.594 | -0.0641 | No |
| 29 | LRP6 | LDL receptor related protein 6 [Source:HGNC Symbol;Acc:HGNC:6698] | 12051 | -0.607 | -0.0048 | No |
| 30 | WNT4 | Wnt family member 4 [Source:HGNC Symbol;Acc:HGNC:12783] | 12331 | -0.686 | 0.0442 | No |
Table: GSEA details [plain text format]

  

Fig 2: GOMF\_FRIZZLED\_BINDING      
 Blue-Pink O' Gram in the Space of the Analyzed GeneSet

  

Fig 3: GOMF\_FRIZZLED\_BINDING: Random ES distribution      
 Gene set null distribution of ES for **GOMF\_FRIZZLED\_BINDING**
